# IL-4 together with IL-1β induces antitumor Th9 cell differentiation in the absence of TGF-β signaling

**DOI:** 10.1038/s41467-019-09401-9

**Published:** 2019-03-26

**Authors:** Gang Xue, Guangxu Jin, Jing Fang, Yong Lu

**Affiliations:** 1 Department of Microbiology & Immunology, Wake Forest School of Medicine, Winston-Salem, NC USA; 2 Department of Cancer Biology, Wake Forest School of Medicine, Winston-Salem, NC USA

## Abstract

IL-9-producing CD4^+^ (Th9) cells are a subset of CD4^+^ T-helper cells that are endowed with powerful antitumor capacity. Both IL-4 and TGF-β have been reported to be indispensable for Th9 cell-priming and differentiation. Here we show, by contrast, that Th9 cell development can occur in the absence of TGF-β signaling. When TGF-β was replaced by IL-1β, the combination of IL-1β and IL-4 efficiently promoted IL-9-producing T cells (Th9^IL-4+IL-1β^). Th9^IL-4+ IL-1β^ cells are phenotypically distinct T cells compared to classic Th9 cells (Th9^IL-4+TGF-β^) and other Th cells, and are enriched for IL-1 and NF-κB gene signatures. Inhibition of NF-κB but not TGF-β-signaling negates IL-9 production by Th9^IL-4+IL-1β^ cells. Furthermore, when compared with classic Th9^IL-4+TGF-β^ cells, Th9^IL-4+IL-1β^ cells are less exhausted, exhibit cytotoxic T effector gene signature and tumor killing function, and exert a superior antitumor response in a mouse melanoma model. Our study thus describes an alternative pathway for Th9 cell differentiation and provides a potential avenue for antitumor therapies.

## Abstract (web summary)

CD4^+^ helper T cells producing IL-9 (Th9) have been implicated in anti-tumor immunity, with Th9 differentiation inducible in vitro via IL-4 and TGFβ treatment. Here the authors show that replacing TGFβ with IL-1β induces a distinct IL-9^+^ CD4^+^ population that have strong cytotoxic and anti-tumor activity in preclinical mouse models.

## Introduction

Interleukin-9 (IL-9)-producing CD4^+^ T helper 9 (Th9) cells are a distinct subset of Th cells induced from naive CD4^+^ T cells by IL-4 together with transforming growth factor-β (TGF-β) cytokine signaling^1,2^. Although Th9 cell differentiation requires a regulatory network of transcription factors and Th9 cells express transcription regulators such as PU.1, IRF4, STAT6, GATA3, BATF, STAT5, HIF1α, and Foxo1^3–10^, a unifying master transcription factor is still ambiguous. In addition to roles in allergic inflammation and autoimmune diseases, the most intriguing function of Th9 cells is their antitumor activity^4,10–12^. We were among the first to report antitumor features of Th9 cells^13^. Furthermore, increased physiological Th9 cell counts during nivolumab (anti-PD-1 antibodies (Abs)) treatment were associated with an improved clinical response among patients with metastatic melanoma^14^. More recently, we reported that Th9 cells represent a novel third paradigm for T cell therapy—they are less exhausted, fully cytolytic, and hyperproliferative, and only tumor-specific Th9 cells completely eradicated late-stage advanced tumors, a scenario more like that seen clinically^15^. Thus further work to elucidate the development of Th9 cells is warranted.

Signals from IL-4 and TGF-β have been recognized as indispensable for Th9 cell differentiation, and neither IL-4 nor TGF-β is sufficient by itself to generate the Th9 cell transcriptional profile or to induce high amounts of IL-9 expression in T cells^6,10,16^. One study showed that Activin A, a member of TGF-β superfamily, may replicate the function of TGF-β in driving in vitro generation of Th9 cells^17^. However, the requirement for TGF-β signaling is unclear; one report has shown that IL-9 production from CD4^+^ T cells during a parasite infection is comparable between wild-type (WT) mice and TGF-βRII dominant-negative mice (which express a dominant-negative TGF-β receptor)^18^. Thus in the current study we sought to identify the potential of other cytokine combinations that may lead to Th9 cell priming and development.

Here we report that Th9 cell differentiation can occur in the absence of TGF-β signaling. IL-4 in combination with IL-1β effectively induces generation of IL-9-producing CD4^+^ T cells (Th9^IL-4+IL-1β^), independent of endogenous TGF-β signaling. We demonstrate that the nuclear factor (NF)-κB pathway is required for IL-9 production in Th9^IL-4+IL-1β^ cells. Furthermore, Th9^IL-4+IL-1β^ cells promote antitumor immune responses in our experimental tumor-bearing model in vivo, achieving superior outcomes than those from classic Th9^IL-4+TGF-β^ cells.

## Results

### IL-4 together with IL-1β induces IL-9-producing CD4^+^ Th9 cells

**Fig. 1:**
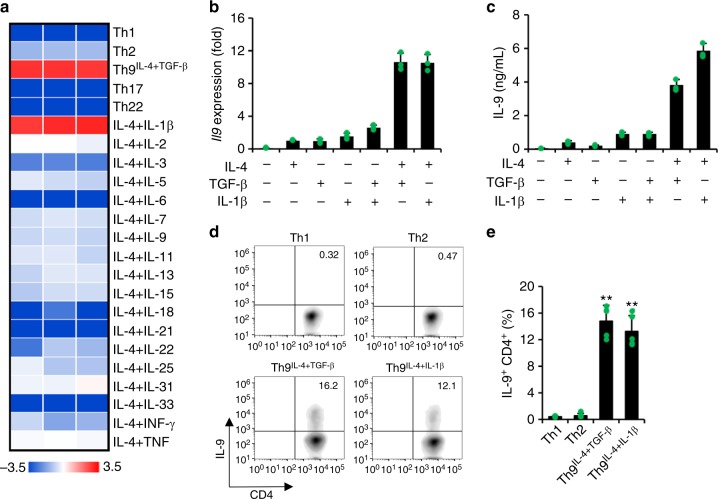
Interleukin (IL)-4 in combination with IL-1β promotes IL-9-producing CD4^+^ T cell development. Naive CD4^+^ CD62L^+^ T cells were purified from the spleens of tyrosinase-related protein-1 mice and cocultured with irradiated antigen-presenting cells under polarized conditions as detailed in the Methods section (polarized in vitro for 3 days). **a** Reverse transcriptase–PCR (RT-PCR) was performed to determine the expression of *Il9* genes. Shown is the heatmap illustrating the relative expression of *Il9* (data are log scaled) (*n* = 3). **b** RT-RCR analysis of *Il9* expression in T cells primed with different cytokines as indicated (*n* = 3). **c** IL-9 production was measured by enzyme-linked immunosorbent assay in the supernatants of differentiated T cells in vitro (*n* = 3). **d**, **e** Intracellular staining showing the percentages of IL-9-producing cells in polarized T helper type 1 (Th1), Th2, classic Th9^IL-4+ TGF-β^, and Th9^IL-4+IL-1β^ cells (*n* = 5). Representative data (**d**) and summarized results (**e**) are shown. Representative results of one from at least two repeated experiments are shown. Data are mean ± SD; **P < 0.01, compared with Th1 or Th2 cells, Student’s *t* test

Classic Th9 cells are induced by IL-4 in combination with TGF-β cytokine signaling. Here we investigated whether TGF-β or IL-4 may be replaced by other cytokines to generate IL-9-producing CD4^+^ T cells. First, we primed naive tyrosinase-related protein (TRP)-1-specific CD4^+^ T cells with TRP-1 peptide-loaded antigen-presenting cells (APCs) by IL-4 in combination with other cytokines; we also generated other Th cell subsets Th1, Th2, Th17, and Th22 and classic Th9^IL-4+TGF-β^ cells as controls. IL-4 plus IL-1β, but not other cytokines, induced a significant amount of *Il9* expression comparable to classic Th9^IL-4+TGF-β^ cells generated under conventional IL-4 and TGF-β conditions (Fig. 1a). We also primed naive TRP-1-specific CD4^+^ T cells by TGF-β in combination with other cytokines. However, only TGF-β incorporated with IL-4 to promote *Il9* gene expression, and no other cytokine appeared to replace the role of IL-4 (Supplementary Figure 1). These results suggest that the new cytokine milieu (IL-4+IL-1β) plays a crucial role and effectively induces IL-9-producing CD4^+^ cells. We further confirm that IL-4, IL-1β, or TGF-β is not sufficient to upregulate IL-9 expression at both the gene (reverse transcriptase–PCR (RT-PCR)) and protein (enzyme-linked immunosorbent assay (ELISA)) levels, whereas IL-4+IL-1β induces IL-9 expression comparable to the classic IL-4+TGF-β cocktail (Fig. 1b, c). The concentration of IL-1β at 10 ng/ml was used thereafter because it is the optimal dose for IL-9 expression in Th9^IL-4+IL-1β^ cells (Supplementary Figure 2). In addition, Th9^IL-4+IL-1β^ and Th9^IL-4+TGF-β^ cells also produce a similar level of IL-10, which has been considered as another cytokine associated with Th9 cells (Supplementary Figure 3). The intracellular staining further supported the IL-9 production in Th9^IL-4+IL-1β^ cells (Fig. 1d, e), whereas Th9^IL-4+IL-1β^ or Th9^IL-4+TGF-β^ cells produced limited IL-4, IL-5, or IL-13 (Supplementary Figure 4). Taken together, IL-4 with IL-1β is an alternative cytokine combination that efficiently induces IL-9-producing CD4^+^ cells.

The generation of Th9^IL-4+IL-1β^ cells is not limited to TRP-1-specific CD4^+^ T cells. We also used ovalbumin (OVA)-specific CD4^+^ T cells (from OT-II mice) and generated a similar IL-9-producing Th9^IL-4+IL-1β^ cells (Supplementary Figure 5). To determine in which cells, T cells and/or APCs, IL-1β signaling is required, we cocultured WT T cells and *Il1r*-deficient APCs or *Il1r*-deficient T cells and WT APCs. As shown in Supplementary Figure 6a, b, *Il1r* deficiency in T cells but not APCs abrogated IL-9 production of Th9^IL-4+IL-1β^ cells. In addition, IL-4+IL-1β is sufficient to induce IL-9 expression in naive CD4^+^ T cells stimulated with plate-bound αCD3/αCD28 mAbs (Supplementary Figure 6c, d). Finally, in mice bearing lung metastatic B16-OVA tumors, *Il1r* deficiency or CD4^+^ T cell-depletion significantly impaired the IL-9 production of leukocytes isolated from the lung tumor tissues (Supplementary Figure 7), suggesting that IL-1β also contributes to the generation of IL-9-secreting T cells in vivo.

### Th9^IL-4+IL-1β^ cells display a distinct gene signature

**Fig. 2:**
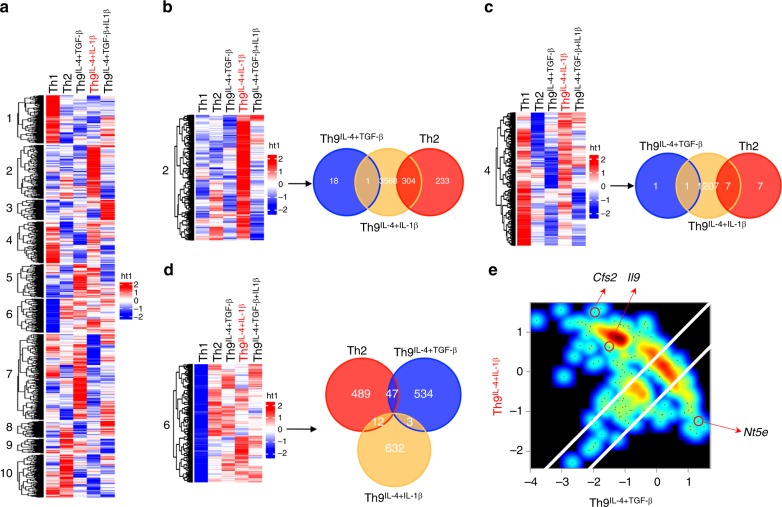
Th9^IL-4+IL-1β^ cells have an identifiable transcriptional signature. Naive CD4^+^ CD62L^+^ T cells were purified from the spleens of tyrosinase-related protein-1 mice and cocultured with irradiated antigen-presenting cells under polarized conditions for 3 days, and RNA was extracted for gene array analysis. **a** Hierarchical clustering of expression levels of 32,471 transcripts in T helper type 1 (Th1), Th2, classic Th9^IL-4+TGF-β^, Th9^IL-4+IL-1β^, and Th9^IL-4+TGF-β+IL-1β^ cells. Cut-tree algorithm divided the transcripts into 10 clusters according to the distances between them defined by average method. **b** Venn diagrams displaying the number of upregulated genes of Th2, classic Th9^IL-4+TGF-β^, and Th9^IL-4+IL-1β^ cells in cluster 2. The threshold for the upregulated genes is set as *z*-score = 0.9. **c** Venn diagrams showing the number of upregulated genes of Th2, classic Th9^IL-4+TGF-β^, and Th9^IL-4+IL-1β^ cells in cluster 4. The threshold for the upregulated genes is set as *z*-score = 0.9. **d** Venn diagrams showing the number of upregulated genes of Th2, classic Th9^IL-4+TGF-β^, and Th9^IL-4+IL-1β^ cells in cluster 6. The threshold for the upregulated genes is set as *z*-score = 0.9. **e** Specific genes upregulated or downregulated by Th9^IL-4+IL-1β^ cells versus classic Th9^IL-4+TGF-β^. Axis denotes the scaled expression levels represented by *z*-scores in the scatter plot. *Csf2* and *Il9* are uniquely upregulated (*z*-score is >1 and 0, respectively) in Th9^IL-4+IL-1β^ cells, whereas *Nt5e* is specifically upregulated in classic Th9^IL-4+TGF-β^ cells with *z*-score >1

Next, we explored whether IL-4- and IL-1β-induced Th9^IL-4+ IL-1β^ cells represent a unique T cell subset compared with other known Th cells. We performed a microarray analysis comparing Th9^IL-4+IL-1β^ cells with Th1, Th2, classic Th9^IL-4+TGF-β^ cells, and Th9^IL-4+TGF-β+IL-1β^ cells (generated in the presence of TGF-β together with IL-4 and IL-1β, in which IL-1β serves a stimulator to further enhance the IL-9 expression from classic Th9^IL-4+TGF-β^ cells^19,20^). Clustering analysis indicated that Th9^IL-4+ IL-1β^ cells have a gene signature that is distinct from any other four types of Th cells (Fig. 2a). Gene clustering also identified three gene clusters (clusters 2, 4, and 6) enriched in these unique genes that are specifically upregulated in Th9^IL-4+IL-1β^ cells. These three clusters were further explored using commonality analysis^21^ and overlap among the three cell types Th2, classic Th9^IL-4+TGF-β^, and Th9^IL-4+IL-1β^ were analyzed by Venn diagrams. We detected 304, 7, and 12 overlapping transcriptional changes between Th9^IL-4+IL-1β^ cells and Th2 cells in clusters 2, 4, and 6, respectively; whereas only 1, 1, and 3 genes in clusters 2, 4, and 6, respectively, were identified as overlapping transcriptional changes between Th9^IL-4+IL-1β^ cells and classic Th9^IL-4+TGF-β^ cells (Fig. 2b–d). Of note, these overlapping genes in clusters 2, 4, and 6 only account for ~about 6% of all unique genes in Th9^IL-4+ IL-1β^ cells, suggesting that they may represent a separate T cell phenotype. Next, we analyzed global gene expression in Th9^IL-4+IL-1β^ and classic Th9^IL-4+TGF-β^ cells. Th9^IL-4+ IL-1β^ cells possess >50% differentially expressed genes compared to classic Th9^IL-4+TGF-β^ cells (Fig. 2e). Apart from increased *Il9* expression, Th9^IL-4+IL-1β^ cells also express markedly increased *Csf2* (the gene that encodes GM-CSF), which is reported to be a unique gene induced by IL-1β^22,23^. By contrast, classic Th9^IL-4+TGF-β^ cells show highly upregulated *Nt5e*, the gene specifically promoted by TGF-β^24,25^. Taken together, these data indicate that Th9^IL-4+IL-1β^ cells have an identifiable transcriptional signature that may result in unique functional properties.

### Th9^IL-4+IL-1β^ cell generation is independent of TGF-β signaling

**Fig. 3:**
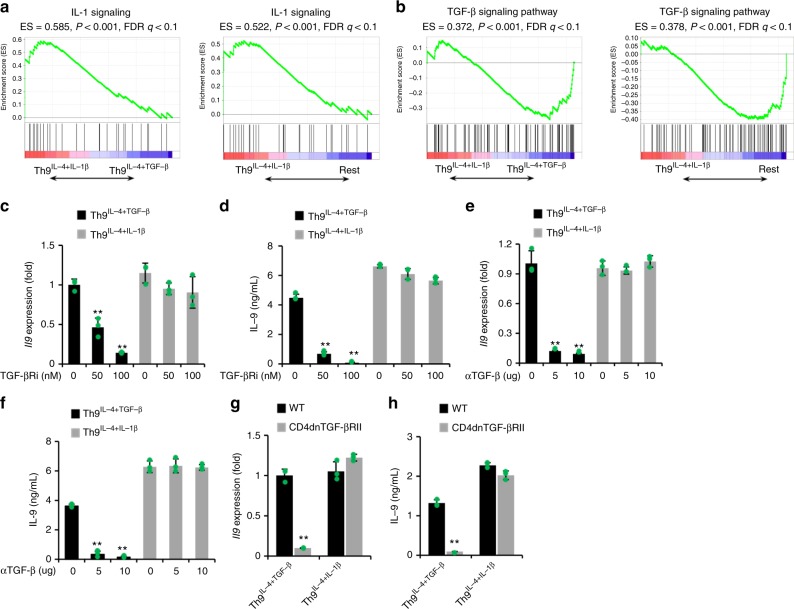
Differentiation of Th9^IL-4+IL-1β^ cells is transforming growth factor (TGF)-β independent. **a**–**f** T cells differentiation and gene array data are the same as shown in Fig. 2. **a** Gene set enrichment analysis (GSEA) of Th9^IL-4+IL-1β^ cells versus classic Th9^IL-4+TGF-β^ cells or the Rest T helper (Th) cells for interleukin (IL)-1 signaling genes. Rest Th cells contain Th1, Th2, Th9^IL-4+TGF-β^, and Th9^IL-4+TGF-β+IL-1β^ cells. **b** GSEA of Th9^IL-4+IL-1β^ versus classic Th9^IL-4+TGF-β^ cells or the Rest Th cells for TGF-β signaling genes. **c**, **d** Reverse transcriptase–PCR (RT-PCR) analysis of *Il9* transcriptional level (**c**) and enzyme-linked immunosorbent assay (ELISA) of IL-9 production in the supernatants (**d**) from TGF-βRi-treated classic Th9^IL-4+TGF-β^ cells or Th9^IL-4+IL-1β^ cells at the indicated concentrations (*n* = 3). TGF-βRi TGF-β receptor serine kinase inhibitor. **e**, **f** RT-PCR analysis of *Il9* transcriptional level (**e**) and ELISA of IL-9 production in the supernatants (**f**) from αTGF-β-treated classic Th9^IL-4+TGF-β^ cells or Th9^IL-4+IL-1β^ cells at the indicated concentrations (*n* = 3). αTGF-β TGF-β monoclonal antibody (mAb). **g**, **h** Naive CD4^+^ CD62L^+^ T cells were purified from the spleens of wild-type (WT) mice or CD4dnTGF-βRII mice and cultured with plate-bound anti-CD3 mAbs and soluble anti-CD28 mAbs under polarized conditions, as detailed in the Methods section, for 3 days. RT-PCR analysis of *Il9* transcriptional level (**g**) and ELISA of IL-9 production in the supernatants (**h**) of classic Th9^IL-4+TGF-β^ cells and Th9^IL-4+IL-1β^ cells from WT mice or CD4dnTGF-βRII mice after in vitro differentiation (*n* = 3). CD4dnTGF-βRII mice mice expressing a dominant-negative TGF-β receptor. Data are mean ± SD; **P < 0.01, Student’s *t* test. Representative results of one from two repeated experiments are shown

Endogenously produced TGF-β may contribute to IL-9 production from Th9^IL-4+IL-1β^ cells. To explore the contribution of endogenous TGF-β signaling, we first analyzed the IL-1β and TGF-β signaling pathway signatures in these Th cells. Using gene set enrichment analysis (GSEA), gene sets defined as in the IL-1-signaling pathway were imprinted in Th9^IL-4+IL-1β^ cells compared to classic Th9^IL-4+TGF-β^ cells and in all other Th cells (including Th1, Th2, and Th9^IL-4+TGF-β+IL-1β^ cells) (Fig. 3a). As expected, TGF-β signaling signature was markedly downregulated in Th9^IL-4+IL-1β^ cells compared to classic Th9^IL-4+TGF-β^ cells or Rest Th cells (Fig. 3b). Thus we hypothesize that TGF-β signaling is not required to produce IL-9 in Th9^IL-4+IL-1β^ cells. To test our hypothesis, we generated Th9^IL-4+IL-1β^ cells in the presence of TGF-β receptor serine kinase inhibitor (TGF-βRi). TGF-βRi treatment significantly downregulated *Il9* gene expression and IL-9 secretion in the classic Th9^IL-4+TGF-β^ cells but did not change IL-9 production in Th9^IL-4+IL-1β^ cells (Fig. 3c, d). To address the off-target potential of a chemical inhibitor, we also used anti-TGF-β antibody or CD4dnTGF-bRII mice. TGF-β neutralization abrogated IL-9 production in classic Th9^IL-4+TGF-β^ cells, while IL-9 production in Th9^IL-4+IL-1β^ cells was not affected (Fig. 3e, f). Similar results were obtained when using naive TGF-βRII^null^ CD4^+^ T cells isolated from CD4dnTGF-βRII mice. IL-9 production was markedly reduced in classic Th9^IL-4+TGF-β^ cells but not in Th9^IL-4+IL-1β^ cells generated from TGF-βRII^null^ CD4^+^ T cells (Fig. 3g, h). To exclude the possibility that IL-1β promotes the production of other TGF-β superfamily^17^ and then indirectly induces IL-9 production, we checked the activation status of Smads in Th9^IL-4+IL-1β^ cells compared to classic Th9^IL-4+TGF-β^ cells. As shown in Supplementary Figure 8, Th9^IL-4+TGF-β^ but not Th9^IL-4+ IL-1β^ cells had activated Smads, and as expected, Smad inhibitors had little impact on IL-9 production in Th9^IL-4+ IL-1β^ cells. Collectively, these results suggested that IL-4 plus IL-1β can effectively induce generation of Th9 cells without TGF-β signaling.

### NF-κB pathway is required for Th9^IL-4+IL-1β^ cell generation

**Fig. 4:**
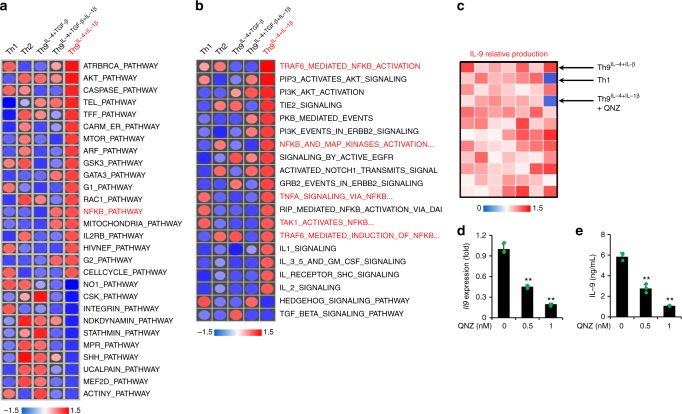
Nuclear factor (NF)-κB pathway is required for Th9^IL-4+IL-1β^ cell differentiation. T cells differentiation and gene array data are the same as shown in Fig. 2. **a**, **b** Heatmaps of enriched signaling of the most upregulated/downregulated pathways for T helper type 1 (Th1), Th2, classic Th9^IL-4+TGF-β^, Th9^IL-4+IL-1β^, and Th9^IL-4+TGF-β+IL-1β^ cells. **c** Interleukin (IL)-9 production was measured by enzyme-linked immunosorbent assay (ELISA) in the supernatants of in vitro differentiated T cells. Shown is the heatmap of IL-9 relative production in the supernatants of Th1 and Th9^IL-4+IL-1β^ cells and inhibitor-treated Th9^IL-4+IL-1β^ cells (Kinase Inhibitor library, also see Supplementary Figure 10a). **d**, **e** RT-PCR analysis of *Il9* transcriptional level (**d**) and ELISA of IL-9 production in the supernatants (**e**) of QNZ-treated Th9^IL-4+IL-1β^ cells at the indicated concentrations (*n* = 3). QNZ NF-κB pathway inhibitor. Data are mean ± SD; **P < 0.01, Student’s *t* test. Representative results from one of two repeated experiments are shown

It is also important to determine what signaling pathway(s) is responsible for IL-9 production from Th9^IL-4+IL-1β^ cells. We first checked *Irf1*, because it has been reported to regulate IL-9 production in Th9^IL-4+TGF-β+IL-1β^ cells^20^. However, we found no difference between IL-9 production in Th9^IL-4+IL-1β^ cells generated from naive CD4^+^ T cells isolated from WT mice or *Irf1*-knockout mice (Supplementary Figure 9). To identify the potential pathway for IL-9 induction, we performed GSEA for enriched signaling(s) in Th9^IL-4+IL-1β^ cells (674 Reactome and 186 KEGG gene sets). As shown in Fig. 4a, b for the most upregulated/downregulated pathways, it seems that The NF-κB and AKT pathways were specifically activated in Th9^IL-4+IL-1β^ cells compared with other Th cells. Using a panel of chemicals containing 82 pathway inhibitors (Supplementary Figure 10a), only the NF-κB pathway inhibitor QNZ significantly suppressed IL-9 production of Th9^IL-4+IL-1β^ cells (Fig. 4c). We further confirmed that inhibition of IL-9 production of Th9^IL-4+IL-1β^ cells by QNZ was dose dependent (Fig. 4d, e), whereas such effect on Th9^IL-4+TGF-β^ cells was moderate in comparison to that of Th9^IL-4+IL-1β^ cells (Supplementary Figure 10b-10d). To further investigate the molecular requirement, we first used a luciferase reporter assay to examine which NF-κB family member(s) can bind and transcribe *Il9* gene. We found RelA but not other members significantly enhanced the *Il9* promoter transcriptional activity (Supplementary Figure 11a). In addition, we detected a significantly increased binding of RelA to the *Il9* promoter in Th9^IL-4+IL-1β^ cells by chromatin immunoprecipitation (ChIP) assay (Supplementary Figure 11b). Finally, knockdown of RelA by small interfering RNA (siRNA) largely reduced IL-9 production in Th9^IL-4+IL-1β^ cells (Supplementary Figure 11c). Therefore, these data suggest that activation of the NF-κB pathway is required for IL-9 production in Th9^IL-4+IL-1β^ cells.

### Th9^IL-4+IL-1β^ cells have a cytolytic effector signature

**Fig. 5:**
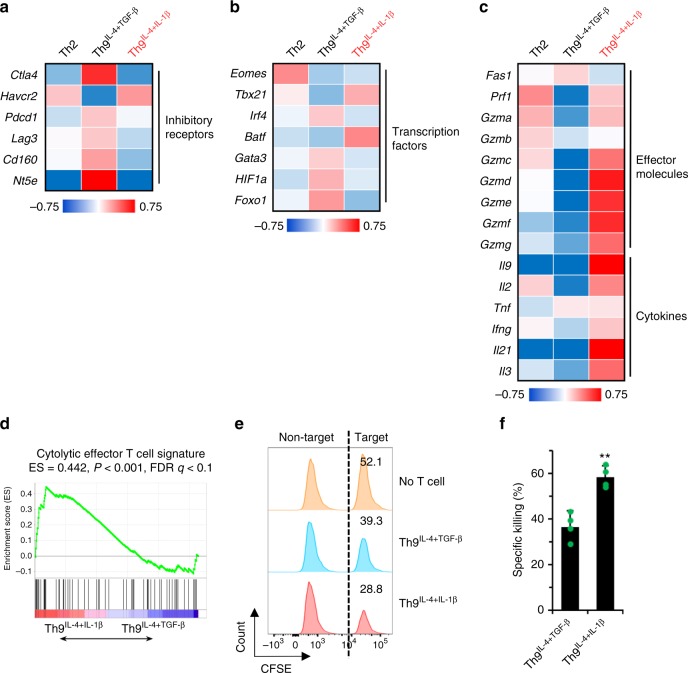
Th9^IL-4+IL-1β^ cells exhibited antitumor activity in vitro. T cells differentiation and gene array data are the same as shown in Fig. 2. **a** Heatmap illustrating the relative expression of exhaustion/inhibition markers as indicated (data are log scaled). **b** Heatmap illustrating the relative gene expression of transcription factors as indicated (data are log scaled). **c** Heatmap illustrating the relative gene expression (data are log scaled). **d** Gene set enrichment analysis of classic Th9^IL-4+TGF-β^ cells versus Th9^IL-4+IL-1β^ cells for cytolytic effector T cell signature. **e**, **f** Specific killing assay of tyrosinase-related protein-1 classic Th9^IL-4+TGF-β^ or Th9^IL-4+IL-1β^ was performed against CFSE^high^-B16 tumor cells as target cells and CFSE^low^-MC38 tumor cells as a non-target control. An E:T ratio of 10:1 was used, and specific killing was determined after 48 h of coculture. Representative data (**e**) and summarized data (**f**) are shown (*n* = 4). Data are mean ± SD; **P < 0.01, Student’s *t* test. Representative results from one of two repeated experiments are shown

Our group has a long-term interest in using tumor-specific Th9 cells as a novel therapeutic strategy for malignancies^7,13,15,26,27^. We next investigated whether these TGF-β signaling-independent Th9^IL-4+IL-1β^ cells are also potent antitumor T cells. Analysis of gene array data suggests that most exhaustion/inhibition markers are downregulated in Th9^IL-4+IL-1β^ cells compared to classic Th9^IL-4+TGF-β^ cells, including *Ctla4*, *Pdcd1*, *Lag3*, and *NT5e* (Fig. 5a). In particular, Th9^IL-4+IL-1β^ cells had greater expression of *Eomes* and *Tbx21* (Fig. 5b), transcriptional factors that suggest effector cell development^28–30^, and increased expression of a *Grz* panel (*Grza*, *Grzb*, *Grzc*, *Grzd*, *Grze*, *Grzf*, *Grzg*) and *Prf1* (Fig. 5c). Enhanced expression of cytokine genes, such as *Il9*, *Il2*, and *Il21*, may also be involved in the superior antitumor functionality of Th9^IL-4+IL-1β^ cells. These observations prompted us to use GSEA to generate an enrichment plot for a cytolytic effector T cell gene signature. GSEA of the gene array data revealed that, as compared with the classic Th9^IL-4+TGF-β^ cells, the cytolytic effector T cell gene signature was significantly enriched in Th9^IL-4+IL-1β^ cells (Fig. 5d), further suggesting that Th9^IL-4+IL-1β^ cells are also potent antitumor effector T cells. Indeed, tumor-specific Th9^IL-4+IL-1β^ cells displayed significantly higher tumor-specific killing activity compared to classic Th9^IL-4+TGF-β^ and Th2 cells in the in vitro cytolytic killing assay (Fig. 5e, f and Supplementary Figure 12a).

### Th9^IL-4+IL-1β^ cells are potent antitumor T cells

**Fig. 6:**
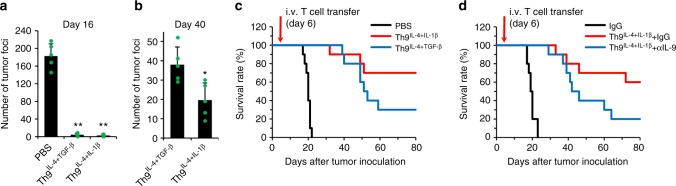
The antitumor activity of Th9^IL-4+IL-1β^ cells in vivo. C57BL/6 mice were challenged with 1 × 10^5^ B16 cells delivered intravenously. T cells were differentiated as in Fig. 2, and 1 × 10^6^ tyrosinase-related protein (TRP)-1 T cells were transferred on day 6 after tumor challenge. **a**, **b** TRP-1-specific classic Th9^IL-4+TGF-β^ cells or Th9^IL-4+IL-1β^ cells were transferred into tumor-bearing mice, tumor foci in the lung were counted on day 16 (**a**) or day 40 (**b**) after tumor inoculation (*n* = 5 mice/group). **c** The survival rate of tumor-bearing mice with the indicated treatments (*n* = 10 mice). **d** The survival rate of tumor-bearing mice with the indicated treatments (*n* = 10 mice). Control IgG or αIL-9 were injected intraperitoneally twice every week starting at 1 day before intravenous transfer of T cells. Data are mean ± SD; *P < 0.05, **P < 0.01, Student’s *t* test. Representative results from one of two repeated experiments are shown

Next, we investigated whether tumor-specific Th9^IL-4+IL-1β^ cells possess antitumor capacity in vivo. We used the TRP-1 model of adoptive immunotherapy, which reproduces the clinical challenge of targeting gp75 tumor/self-antigen in the poorly immunogenic B16 melanoma model^15^. We transferred TRP-1-specific Th2 cells or classic Th9^IL-4+TGF-β^ cells or Th9^IL-4+IL-1β^ cells into B6 mice bearing 6-day established B16 lung metastatic murine melanoma. Analyzing the lung tumor foci on 16 days after tumor inoculation suggested that Th9^IL-4+IL-1β^ cells induced robust tumor clearance compared to phosphate-buffered saline (PBS) or Th2 cell-treated groups (Fig. 6a and Supplementary Figure 12b), and Th9^IL-4+IL-1β^ cells also showed superior long-term (40 days) antitumor capacity compared to classic Th9^IL-4+TGF-β^ cells (Fig. 6b). In addition, on day 80, 70% of Th9^IL-4+IL-1β^ cell-treated mice were alive versus 30% of mice receiving the classic Th9^IL-4+TGF-β^ cells (Fig. 6c). Finally, IL-9 production from Th9^IL-4+IL-1β^ cells contributed to antitumor efficacy, since neutralization of IL-9 reduced survival of Th9^IL-4+IL-1β^ cell-treated mice (Fig. 6d). Overall, our study indicates that Th9^IL-4+IL-1β^ cells are less exhausted T cells endowed with a cytotoxic T effector gene signature that can exert a superior antitumor response compared to classic Th9^IL-4+TGF-β^ cells.

## Discussion

IL-4 and TFG-β have long been considered as the two essential cytokines to induce Th9 cell differentiation. Although additional cytokines (e.g., type I interferon (IFN), IL-1, IL-2, and IL-25)^9,19,20,31,32^ and costimulatory signaling (e.g., OX40 and GITR)^33–35^ enhance IL-9 production and Th9 cell differentiation, the studies were performed in the context of the standard cytokine milieu containing IL-4 and TGF-β. No study has thus far investigated whether IL-4 or TGF-β is replaceable for Th9 induction. In the current study, we, for the first time, found that Th9 cell development can occur in the absence of TGF-β signaling. When TGF-β has been replaced by IL-1β, differentiation of IL-9-producing T cells can be achieved. The presence of APCs may further facilitate Th9^IL-4+IL-1β^ cell differentiation, which may be a synergistic effect of APC-induced costimulation upon IL-1β stimulation. Our study provides new insight into Th9 cell development and may be crucial to understanding the signaling pathways that regulate Th9 cell fate and functions.

Our new Th9^IL-4+IL-1β^ cells may represent a completely separate T cell phenotype compared to classic Th9^IL-4+TGF-β^ cells and other known Th subsets (Th1 and Th2 cells). This is supported by our gene array analysis of the global gene profile of Th9^IL-4+IL-1β^ cells. We distinguished 3 gene clusters with key gene features of Th9^IL-4+IL-1β^ cells, within which only ~6% of the unique genes are shared with Th2 or classic Th9^IL-4+TGF-β^ cells. When directly compared with the classic Th9^IL-4+TGF-β^ cells, the new Th9^IL-4+IL-1β^ cells display a large portion of differentially expressed genes. As expected, Th9^IL-4+IL-1β^ cells are imprinted with the IL-1 signaling pathway signature while the classic Th9^IL-4+TGF-β^ cells are enriched in the TGF-β signaling signature; this may be caused by the different priming cytokine signaling from IL-1β versus TGF-β. Our study also excluded the possibility that endogenously produced TGF-β or endogenous TGF-β superfamily member signaling in naive CD4^+^ T cells contribute to IL-9 production in Th9^IL-4+IL-1β^ cells. These studies indicate Th9^IL-4+IL-1β^ cells are phenotypically different from TGF-β-dependent classic Th9^IL-4+TGF-β^ cells.

For development of classic Th9^IL-4+TGF-β^ cells, a large number of signaling molecules has been reported to be crucial for IL-9 transcription, including those downstream of IL-4 signaling (e.g., signal transducer and activator of transcription factor 6 (STAT6) and GATA3), TGF-β signaling (e.g., PU.1, TAK1, HIF1α, and SMAD), and common signaling/transcriptional factors in T cells (e.g., IRF4, BATF, STAT5, AKT, FOXO1, NFAT, and Notch)^7,8,10,36^. Thus it appears that IL-9 production or Th9^IL-4+TGF-β^ cell differentiation is not regulated by a master transcription factor but instead by a coordinated network. Using GSEA, we identified markedly enriched NF-κB and AKT pathway signatures in Th9^IL-4+IL-1β^ cells. After adding a panel of individual inhibitors at the beginning of the Th9^IL-4+IL-1β^ cell priming, we confirmed that IL-9 production depended on NF-κB. It has been reported that a variety of immunologically relevant ligands, like IL-1 family members, TNF family members, etc., can activate NF-κB pathway^37^. Our results suggest that NF-κB family member RelA is required for IL-9 production in Th9^IL-4+ IL-1β^ cells. However, RelB/p52 has been reported to be more active than RelA/p50 for IL-9 production in Th9^IL-4+TGF-β^ cells upon OX40 costimulation^33^. This may be a result of different epigenetic changes of *Il9* gene promoter region in Th9^IL-4+IL-1β^ versus Th9^IL-4+TGF-β^ cells, which allows unique binding accessibilities for different NF-κB family members. Thus signaling through the NF-κB pathway, mainly RelA, is essential for *Il9* transcription and IL-9 production in the Th9^IL-4+ IL-1β^ cells.

Our long-term interest in Th9 cells is to explore their potential for cancer treatment^7,13,15,26,27^. Thus we investigated whether Th9^IL-4+IL-1β^ cells are also tumor killers. Interestingly, these cells display attractive antitumor effector T cell features, such as further downregulated expression of exhaustion markers and high enrichment in cytolytic effector T cell gene signatures, compared to classic Th9^IL-4+TGF-β^ cells. Direct comparisons of these two phenotypically distinct Th9 cells suggests that Th9^IL-4+IL-1β^ cells display higher tumor-specific cytotoxicity in vitro and improved tumor clearance in vivo compared to classic Th9^IL-4+TGF-β^ cells. Of note, Th9^IL-4+IL-1β^ cell-mediated therapeutic antitumor effects also require IL-9 production. Therefore, our study indicates that Th9^IL-4+IL-1β^ cells are less exhausted cytotoxic effector T cells and can exert an antitumor response superior to classic Th9^IL-4+TGF-β^ cells.

In summary, we report for the first time that TGF-β is not indispensable for Th9 cell differentiation. IL-4 plus IL-1β effectively induced IL-9 production independently of TGF-β signaling. Our study further showed that the NF-κB pathway, mainly RelA, is required for IL-9 production of Th9^IL-4+IL-1β^ cells. Noticeably, tumor-specific Th9^IL-4+IL-1β^ cells are cytolytic effectors and endowed with robust antitumor function. Therefore, our findings confirm the current understanding underlying Th9 cell differentiation and provide an avenue for a potentially more powerful weapon for cancer immunotherapy.

## Methods

### Mice

C57BL/6, B6.Cg-Tg(Cd4-TGFBR2)16Flv/J, B6.129S2-*Irf1*^*tm1Mak*^/J, B6.Cg-Tg(TcraTcrb)425Cbn/J, B6.129S7-*Il1r1*^*tm1Imx*^/J, and B6.Cg-*Rag1*^*tm1Mom*^ *Tyrp1*^*B-w*^ Tg(Tcra,Tcrb)9Rest/J mice were purchased from The Jackson Laboratory. Male and female 6–8-week-old mice were used for each animal experiment. All experiments complied with protocols approved by the Institutional Animal Care and Use Committee at the Wake Forest School of Medicine.

### Cell lines

B16 melanoma cell line was purchased from ATCC; MC-38 cell line was a gift from Dr.Patrick Hwu, MD Anderson Cancer Center. Cells were cultured in Iscove’s Modified Dulbecco’s Medium (Invitrogen) or RPMI 1640 Medium (Invitrogen) supplemented with 10% heat-inactivated fetal bovine serum (ThermoFisher Scientific), 100 U/ml penicillin-streptomycin, and 2 mM L-glutamine (both from Invitrogen).

### Reagents

TGF-β receptor serine kinase inhibitor TGF-βRi (Cat#616452) was purchased from Millipore. NF-κB inhibitor QNZ (Cat#sc-200675) was purchased from Santa Cruz Biotechnology. Smad3 inhibitor SIS3 (Cat#S7959) and Smad2 inhibitor LY2109761 (Cat#S2704) were purchased from Selleckchem. The major histocompatibility complex (MHC) class II-restricted TRP-1^106–130^ peptides (SGHNCGTCRPGWRGAACNQKILTVR) and the MHC class II-restricted OT II^323–339^ peptides (ISQAVHAAHAEINEAGR) were purchased from GenScript. Mouse IL-4-neutralizing antibody (clone 11B11), mouse IFN-γ-neutralizing antibody (clone XMG1.2), mouse IL-9-neutralizing antibody (clone 9C1), mouse CD4 depletion antibody (clone GK1.5), and TGF-β-neutralizing antibody (clone 1D11.16.8) were purchased from BioXcell. Mouse cytokine IL-1β, IL-4, IL-6, IL-12, and TNF and human IL-2 and TGF-β were purchased from R&D Systems. Chemical inhibitor Panel (Enzo, SCREEN-WELL® Kinase Inhibitor library, Cat#BML-2832-0100; also see Supplementary Figure 7a).

### In vitro Th cell differentiation

Naive CD4^+^ CD62L^+^ T cells were purified from the spleens of TRP-1 or OT II mice by isolation kit (STEMCELL Technologies, Cat#19765) according to the manufacturer’s protocol. TRP-1-specific naive CD4 T cells were cultured for 3 days with irradiated T cell-depleted splenic APCs from C57BL/6 mice in the presence of TRP-1^106-130^ peptide (5 μg/ml) or OT II^323–339^ peptides (5 μg/ml). Th1 polarized medium was supplemented with IL-2 (100 U/ml), IL-12 (5 ng/ml), and anti-IL-4 mAbs (10 μg/ml). Th2 polarized medium was supplemented with IL-2 (100 U/ml), IL-4 (10 ng/ml) and anti-IFN-γ mAbs (10 μg/ml). Th17 polarized medium was supplemented with IL-6 (30 ng/ml), TGF-β (2.5 ng/ml), and anti-IFN-γ mAbs (10 μg/ml). Th22 polarized medium was supplemented with IL-6 (10 ng/ml), TNF (5 ng/ml), and anti-IFN-γ mAbs (10 μg/ml). Th9^TGF-β+IL-4^ polarized medium was supplemented with IL-4 (10 ng/ml), TGF-β (1 ng/ml), and anti-IFN-γ mAbs (10 μg/ml). Th9^IL-4+TGF-β+IL-1β^ polarized medium was supplemented with IL-4 (10 ng/ml), TGF-β (1 ng/ml), IL-1β (10 ng/ml), and anti-IFN-γ mAbs (10 μg/ml). Th9^IL-4+IL-1β^ polarized medium was supplemented with IL-4 (10 ng/ml), IL-1β (10 ng/ml, or used at the indicated concentrations), and anti-IFN-γ mAbs (10 μg/ml). In some experiments, naive CD4^+^CD62L^+^ T cells were activated as indicated in the polarized condition with plate-bound anti-CD3 mAbs (1.5 μg/ml, clone 17A2, eBioscience) and anti-CD28 mAbs (0.75 μg/ml, clone 37.51, eBioscience). In some experiments, antibody or inhibitor was added at the beginning of the T cell cultures.

### Real-time PCR

Total RNA was extracted from cultured T cells using the RNeasy Mini Kit (Qiagen), followed by cDNA synthesis with the High-Capacity cDNA Reverse Transcription Kit (Applied Biosystems). RT-PCR was conducted with SYBR Select Master Mix (Applied Biosystems). Expression was normalized to the expression of the housekeeping gene *Gapdh*. *Il9* forward: 5’-ATGTTGGTGACATACATCCTTGC-3’, *Il9* reverse: 5’-TGACGGTGGATCATCCTTCAG-3’; *Irf1* forward: 5’-AGGCATCCTTGTTGATGTCC-3’, *Irf1* reverse: 5’-AATTCCAACCAAATCCCAGG-3’; and *Gapdh* forward: 5’-TTGATGGCAACAATCTCCAC-3’, *Gapdh* reverse: 5’-CGTCCCGTAGACAAAATGGT-3’.

### Flow cytometry

After culture for 3 days, differentiated Th cells were restimulated for 5 h with TRP-1 peptide-loaded APC or phorbol myristate acetate (50 ng/ml; Sigma-Aldrich) and ionomycin (500 ng/ml; Sigma-Aldrich) in the presence of a protein transport inhibitor (GolgiPlug, BD Biosciences). Then cell surface markers were stained in flow cytometry staining buffer for 30 min on ice after Fc blocking, followed by staining for intracellular cytokines with the BD Fixation/Permeabilization Solution Kit. The results were analyzed using a fluorescence-activated cell sorter (FACS) Fortessa flow cytometer. αCD4 (clone RM4-4, 1:100), αIL-9 (clone RM9A4, 1:100), αIL-4 (clone 11B11, 1:100), and αIL-10 (clone JES5-16E3, 1:100) were from BioLegend; αIL-13 (clone eBio13A, 1:100) from ThermoFisher; αp-Smad-3 (clone O72-670, 1:50) from BD; and αp-Smad-5 (clone 41D10, 1:800) from CST.

### Enzyme-linked immunosorbent assay

After 72 h of polarization, cell culture supernatants were tested by ELISA for mouse IL-9 (ThermoFisher) or IL-10 (ThermoFisher) or IL-5 (ThermoFisher) according to the manufacturer’s protocol.

### RNA interference of RelA

siRNA targeting RelA and non-specific siRNA were purchased from ThermoFisher and transfected into T cells by Lipofectamine RNAiMAX Transfection Reagent (ThermoFisher)^38^.

### Luciferase reporter assay

Mouse *Il9* promoter (from −895 to +5) was subcloned into **pGL4.10** basic vector (Promega). 293T cells were transiently transfected 24 h with reporter plasmids along with expression vectors^15^ for RelA, RelB, c-Rel, p50, and p52 or control vector by Lipofectamine 2000 (Invitrogen). Luciferase was measured with the Dual-Luciferase Reporter Assay System according to the manufacturer’s instructions (Promega).

### ChIP assay

SimpleChIP Plus Enzymatic Chromatin IP kits (Cell Signaling Technology) were used for ChIP assays according to the manufacturer’s protocol. For the ChIPn, anti-RelA and isotype-matched control antibody were purchased from Cell Signaling and used at a 1:50 dilution. The DNAs were analyzed by RT-PCR with the following primer set surrounding the RelA-binding site at the *Il9* promoter region. ChIP primers: Forward: 5’-GCACTGGGTATCAGTTTGATG-3’, Reverse: 5’-CTCAGTCTACCAGCATCTTCC-3’. Values were subtracted from the amount of IgG control and were normalized to the corresponding input control.

### Fractionation of the lung

C57BL/6 WT mice or *Il1r* KO mice were challenged with 1 × 10^5^ B16-OVA melanoma cells. Mice were intraperitoneally (i.p.) given control IgG or αCD4-depletion mAbs (clone GK1.5, 200 μg/mice) on days 8 and 11 and then sacrificed on day 14. Lung tumor tissues were digested with 1 mg/ml of collagenase D (Roche) for 30 min at 37 °C and then with 0.01 mM EDTA (Sigma-Aldrich) for 5 min, to prevent aggregates. The cells were collected using Ficoll (GE Healthcare) density gradient centrifugation and the middle section of the gradient was enriched for leukocytes.

### In vitro cytotoxicity assay

B16 target cells for TRP-1 CD4^+^ T cells were labeled with 5 µM carboxyfluorescein succinimidyl ester (CFSE), whereas MC38 nontarget cells were labeled with 0.25 µM CFSE as the control. B16 target (CFSE^high^) or MC38 nontarget (CFSE^low^) cells were incubated with PBS control, TRP-1-specific Th2 cells, TRP-1-specific classic Th9^IL-4+TGF-β^ cells, or TRP-1-specific Th9^IL-4+IL-1β^ cells. An E:T ratio of 10:1 was used, and specific killing was determined after 48 h of coculture. After coculture, CFSE^+^ cells from each target and control well were mixed and analyzed by FACS. The percentage of specific lysis was calculated as follows: (1 − *R* × M2/M1) × 100, where M1 = non-target events, M2 = target events, and R = M1 in untreated group/M2 in untreated group.

### Tumor model and adoptive transfer

To establish the 6-day established murine lung metastatic B16 melanoma model, mice were injected intravenously (i.v.) with 1 × 10^5^ B16 tumor cells. For adoptive transfer experiments, mice were injected i.v. with 1 × 10^6^ TRP-1-specific Th2, Th9^IL-4+TGF-β^, or Th9^IL-4+IL-1β^ cells 6 days after tumor cell inoculation. Control IgG or α-IL-9 were injected i.p. twice every week starting at 1 day before i.v. transfer of T cells. At days 16 or 40 after tumor inoculation, mice were sacrificed for measurement of metastatic lung foci. All lung lobes were evaluated under a tissue-dissecting microscope.

Mice were euthanized by using CO_2_ asphyxiation and followed by cervical dislocation if any of the following symptoms appear: (1) weight loss of ≥20% of baseline body weight; (2) mice display significant lameness and disability in moving.

### Microarray analysis

Total RNA was extracted with the RNeasy Mini Kit (Qiagen) from 3-day cultured Th1, Th2, classic Th9^IL-4+TGF-β^, Th9^IL-4+IL-1β^, and Th9^IL-4+TGF-β+IL-1β^ cells. RNA samples were sent to the Case Western Reserve University Genomic Core for quality evaluation using an Agilent Bioanalyzer. Samples with intact 18S and 28S ribosomal RNA bands with RNA integrity number >8.5 were processed for microarray analysis performed with a Mouse Gene 2.0 ST Kit at the Case Western Reserve University Genomic Core. GSEA was implemented using newly generated gene expression profiles of the five cell types and downloaded GSEA software (gsea-v3.0, http://software.broadinstitute.org/gsea/downloads.jsp). *P* values were calculated with Kolmogorov–Smirnov test (threshold = 0.01). The false discovery rate, *q* value, is the estimated probability that a gene set with a given NES represents a false positive finding. The threshold for *q* value in GSEA is 0.25. Gene sets for the mature effector were derived from a publicly available study of the genes differentially expressed by more than two-fold in quaternary versus primary cells^39^.

### Venn diagram analysis based on Cut-tree algorithm

To compare our Th9^IL-4+IL-1β^ cells with other cells, including known Th9 (Th9^IL-4+TGF-β^), and another Th9 cell induced by three molecules (Th9^IL-4+TGF-β+IL-1β^), and Th1 and Th2 cells, we utilized un-supervised hierarchical clustering^39^ to cluster all genes in the microarray data into 10 clusters. Clusters 1–10 were identified by Cut-tree algorithm using the same distance method of average as hierarchical clustering^39^. Clusters 2, 4, and 6 showing Th9^IL-4+IL-1β^-specific genes (for gene lists, see Supplementary data 1, 2, 3) were taken for commonality analysis by Venn graph^21^. The overlaps among the three cell types Th2, classic Th9^IL-4+TGF-β^, and Th9^IL-4+IL-1β^ were analyzed by Venn diagram. The threshold for the upregulated genes for clusters 2, 4, and 6 is *z*-score = 0.9, where *z*-score is the scaled value of each gene across the 5 cell types. Hierarchical clustering was analyzed by complexheatmap using R and Venn diagram analysis was implemented by VennDiagram in R (https://www.r-project.org/).

### Statistical analyses

For statistical analysis, Student’s *t* test was used. A *P* value <0.05 was considered statistically significant. Results are presented as mean ± standard deviations (SD).

### Reporting Summary

Further information on experimental design is available in the Nature Research Reporting Summary linked to this article.

## Supplementary information

Supplementary Information

Reporting Summary

Description of Additional Supplementary Files

Supplementary Data 1

Supplementary Data 2

Supplementary Data 3

Source Data

## Data Availability

The microarray data reported in this paper are deposited in the Gene Expression Omnibus (GEO) database under the accession number GSE123624. The data for the bar charts and graphs are available in the Source Data file. The other data that support the findings of this study are available from the corresponding author upon reasonable request.
