# Supplementary material for: IL-4 together with IL-1β induces antitumor Th9 cell differentiation in the absence of TGF-β signaling
Source: Nat Commun. 2019 Mar 26;10:1376. doi: 10.1038/s41467-019-09401-9 (PMC6435687; doi:10.1038/s41467-019-09401-9)
Supplement: Supplementary file 1 — Supplementary Information [file 41467_2019_9401_MOESM1_ESM.pdf]

SUPPLEMENTARY INFORMATION

Title: IL-4 together with IL-1β induces antitumor Th9 cell differentiation in the absence of TGF-β signaling

Authors: Gang Xue et al

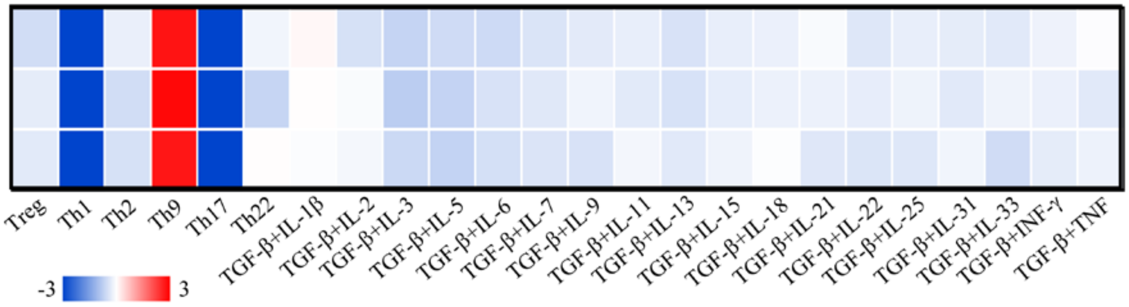

Supplementary Figure 1. Heatmap illustrating the relative expression of *Il9* under conditions as indicated at day 3 after *in vivo* differentiation (data are log scaled). Naïve $CD4^+$ $CD62L^+$ T cells were purified from the spleens of TRP-1 mice and cocultured with irradiated antigen-presenting cells under polarized conditions as detailed in the Methods (polarized *in vitro* for 3 days, n = 3). RT-PCR was used to determine relative *Il9* gene expression. Representative results from one of two repeated experiments are shown.

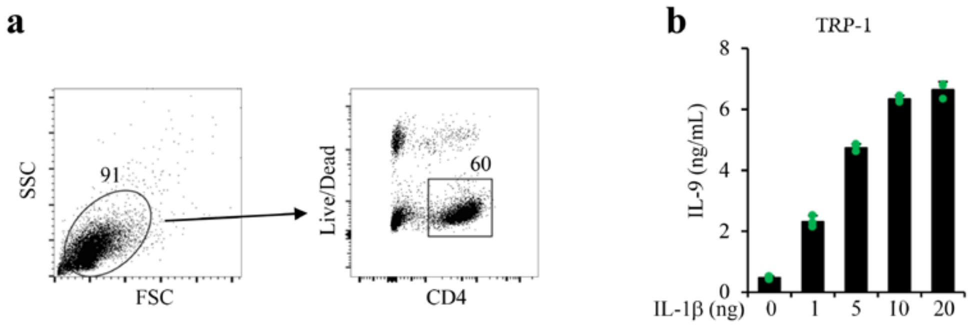

Supplementary Figure 2. ELISA of IL-9 production in the supernatants of Th9$^{IL\text{-}4+IL\text{-}1\beta}$ cells at indicated IL-1β concentrations. (a) Gating strategy to determine $CD4^+$ T cells. (b) Naïve $CD4^+$ $CD62L^+$ T cells were purified from the spleens of TRP-1 mice and cocultured with irradiated antigen-presenting cells under polarized conditions as detailed in the Methods. Data are mean ± SD (polarized *in vitro* for 3 days, n = 3). Representative results from one of two repeated experiments are shown.

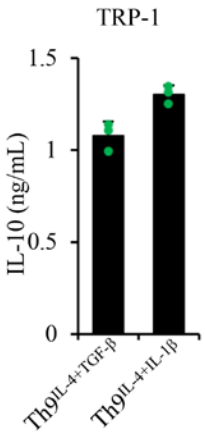

Supplementary Figure 3. ELISA of IL-10 production in the supernatants of Th9$^{IL\text{-}4+TGF\text{-}\beta}$ cells and Th9$^{IL\text{-}4+IL\text{-}1\beta}$ cells. Naïve CD4$^+$ CD62L$^+$ T cells were purified from the spleens of TRP-1 mice and cocultured with irradiated antigen-presenting cells under polarized conditions as detailed in the Methods. Data are mean ± SD (polarized *in vitro* for 3 days, n = 3). Representative results from one of two repeated experiments are shown.

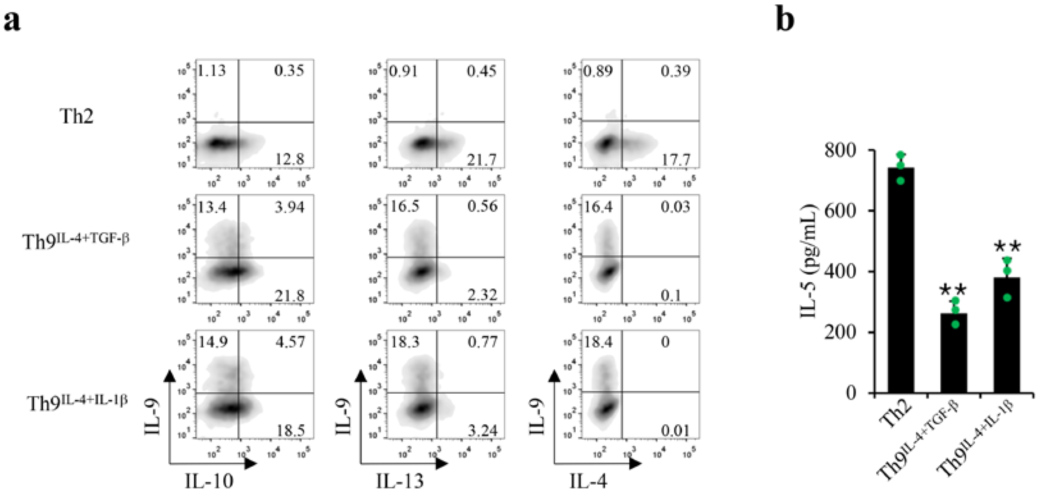

Supplementary Figure 4. The expression of IL-4, IL-5 or IL-13 in Th2, Th9$^{\text{IL-4+TGF-}\beta}$, and Th9$^{\text{IL-4+IL-1}\beta}$ cells. Naïve CD4^{+} CD62L^{+} T cells were purified from the spleens of TRP-1 mice and stimulated with αCD3/αCD28 as detailed in the Methods. (a) Intracellular staining showing the percentages of IL-4, IL-9, IL-10, IL-13 producing cells in polarized Th2, classic Th9$^{\text{IL-4+TGF-}\beta}$ and Th9$^{\text{IL-4+IL-1}\beta}$ cells. (b) ELISA of IL-5 production in the supernatants of Th2, Th9$^{\text{IL-4+TGF-}\beta}$ cells, and Th9$^{\text{IL-4+IL-1}\beta}$ cells. Data are mean ± SD (polarized *in vitro* for 3 days, n = 3). $^{**}P$ <0.01, compared with Th2 cells, Student's *t*-test. Representative results from one of two repeated experiments are shown.

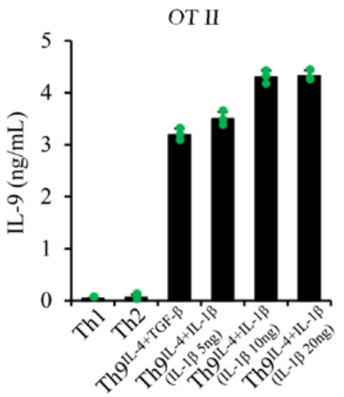

Supplementary Figure 5. ELISA of IL-9 production in the supernatants of OVA-specific Th1, Th2, classic Th9$^{IL\text{-}4+TGF\text{-}\beta}$ and Th9$^{IL\text{-}4+IL\text{-}1\beta}$ cells. Naïve CD4^{+} CD62L^{+} T cells were purified from the spleens of OT II mice and cocultured with irradiated antigen-presenting cells under polarized conditions as detailed in the Methods. Data are mean ± SD (polarized *in vitro* for 3 days, n = 3). Representative results from one of two repeated experiments are shown.

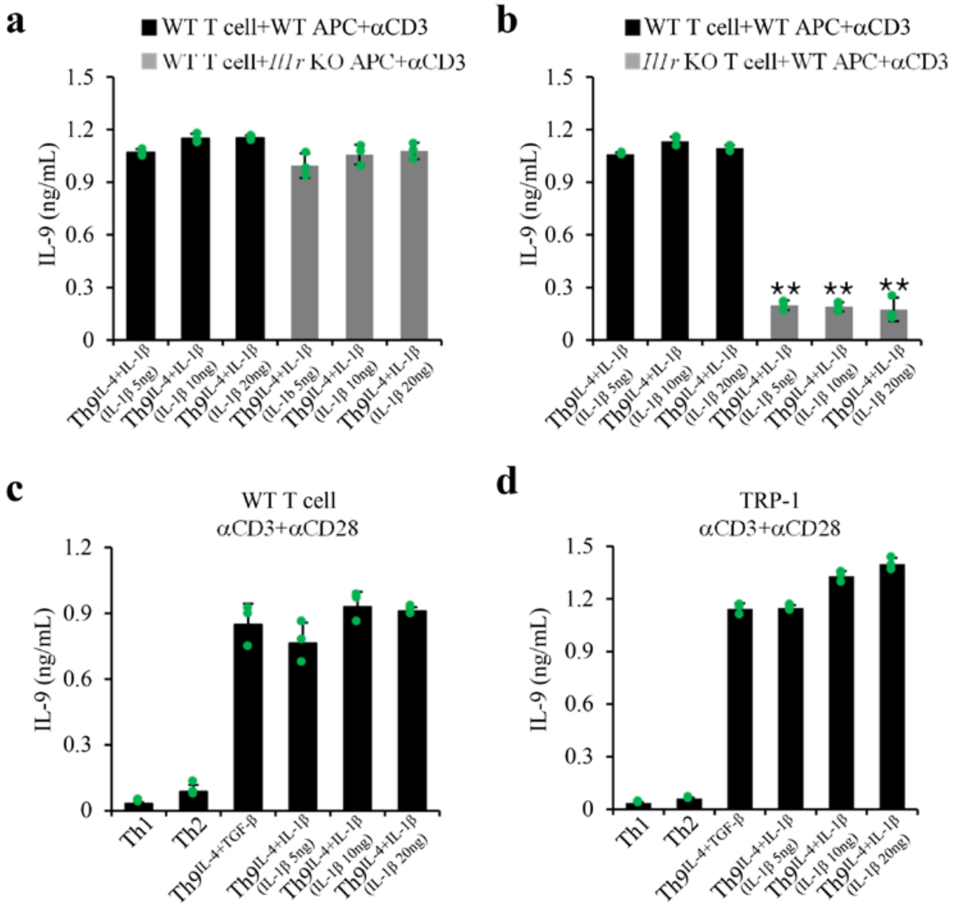

Supplementary Figure 6. ELISA for IL-9 production in the supernatants of Th1, Th2, classic Th9$^{IL\text{-}4+TGF\text{-}\beta}$ and Th9$^{IL\text{-}4+IL\text{-}1\beta}$ cells. (a) Naïve CD4^{+} CD62L^{+} T cells were purified from the spleens of WT mice and cocultured with irradiated antigen-presenting cells from WT mice or *Il1r* KO mice under polarized conditions as detailed in the Methods for 3 days. (b) Naïve CD4^{+} CD62L^{+} T cells were purified from the spleens of WT mice or *Il1r* KO mice and cocultured with irradiated antigen-presenting cells from WT mice under polarized conditions as detailed in the Methods for 3 days. (c) Naïve CD4^{+} CD62L^{+} T cells were purified from the spleens of WT mice and stimulated by plate-bound αCD3/αCD28 under polarized conditions as detailed in the Methods for 3 days. (d) Naïve CD4^{+} CD62L^{+} T cells were purified from the spleens of TRP-1 mice and stimulated by plate-bound αCD3/αCD28 under polarized conditions as detailed in the Methods for 3 days. Data are mean ± SD (polarized *in vitro* for 3 days, n = 3). ***P* <0.01, compared to the cultures with WT T cells, Student's *t*-test. Representative results from one of two repeated experiments are shown.

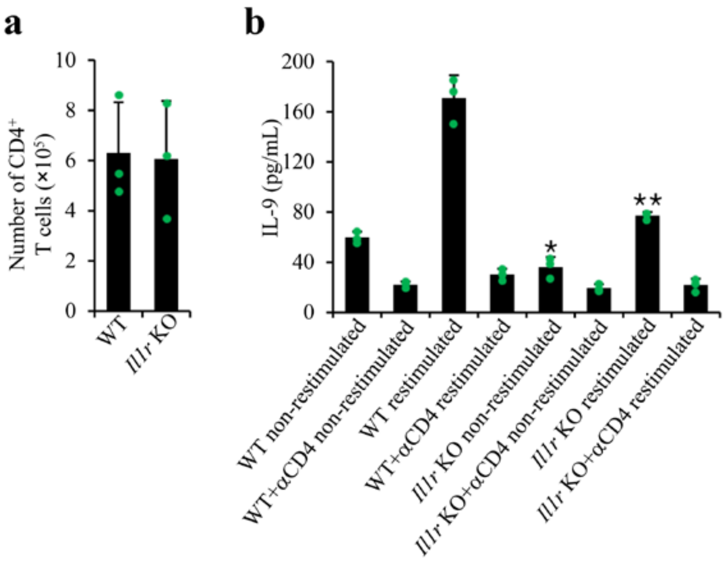

Supplementary Figure 7. IL-9 production was measured by ELISA in the supernatants of restimulated or non-restimulated of mice lung leukocytes. (a) The calculated number of $CD4^+$ T cells in the WT mice and *Il1r* KO mice from lung tumor leukocyte fraction. (b) C57BL/6 mice WT mice or *Il1r* KO mice were challenged with 1×10^5 B16-OVA melanoma cells. Mice were given control IgG or αCD4 mAbs (clone GK1.5, 200 μg/mice) on day 8 and day 11, and then sacrificed on day 14. The lung tumor tissue leukocytes were restimulated or non-restimulated with OT II peptides for 36 hours, and then IL-9 production was measured by ELISA. Data are mean ± SD (n = 3 mice). *$P < 0.05$, compared with WT non-restimulated, **$P <0.01$, compared with WT restimulated, Student's *t*-test. Representative results from one of two repeated experiments are shown.

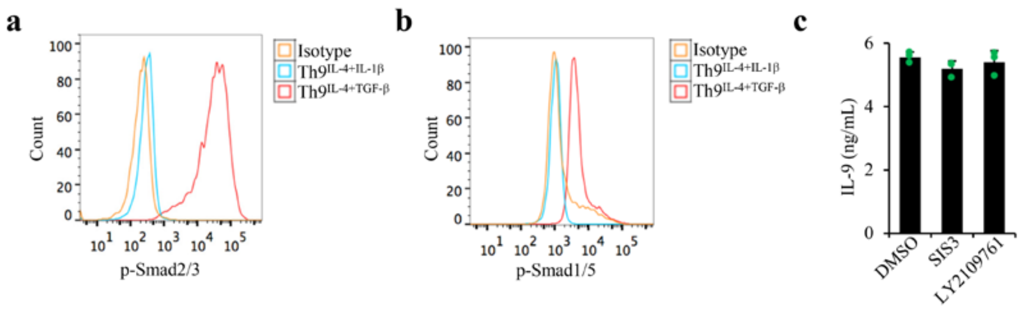

Supplementary Figure 8. Smads activation is not required for IL-9 production in Th9$^{IL\text{-}4+IL\text{-}1β}$ cells. Naïve CD4$^+$ CD62L$^+$ T cells were purified from the spleens of TRP-1 mice and cocultured with irradiated antigen-presenting cells under polarized conditions as detailed in the Methods. (a) Flow analysis of activation status of p-Smad2/3 in Th9$^{IL\text{-}4+IL\text{-}1β}$ cells and Th9$^{IL\text{-}4+TGF\text{-}β}$ cells. (b) Flow analysis of activation status of p-Smad1/5 in Th9$^{IL\text{-}4+IL\text{-}1β}$ cells and Th9$^{IL\text{-}4+TGF\text{-}β}$ cells. (c) ELISA of IL-9 production in the supernatants of Th9$^{IL\text{-}4+IL\text{-}1β}$ cells in the presence of Smads inhibitors or DMSO. Data are mean ± SD (polarized *in vitro* for 3 days, n = 3). Representative results from one of two repeated experiments are shown.

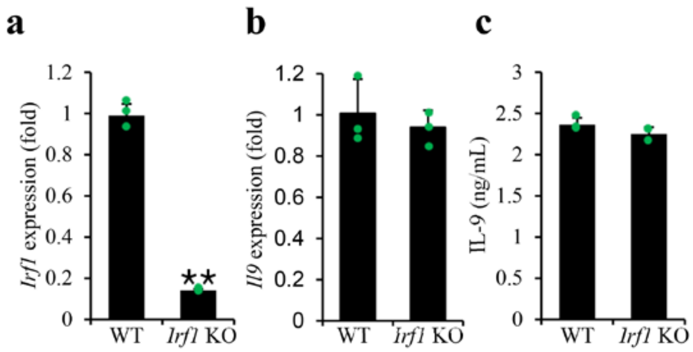

Supplementary Figure 9. IRF-1 is not required for IL-9 production from Th9$^{IL\text{-}4+IL\text{-}1\beta}$ cells. Naïve CD4$^+$ CD62L$^+$ T cells were purified from the spleens of wild-type (WT) mice or *Irf1* knockout (KO) mice and cultured with plate-bound anti-CD3 mAbs and soluble anti-CD28 mAbs under polarized conditions as detailed in the Methods. (a) RT-PCR analysis of the *Irf1* transcriptional level of Th9$^{IL\text{-}4+IL\text{-}1\beta}$ cells from WT mice or *Irf1* KO mice. (b and c) RT-PCR analysis of *Il9* transcriptional level (b) and ELISA of IL-9 production in the supernatants (c) of Th9$^{IL\text{-}4+IL\text{-}1\beta}$ cells from WT mice or *Irf1* KO mice. Data are mean ± SD (polarized *in vitro* for 3 days, n = 3). $^{**}P < 0.01$, Student's *t*-test. Representative results from one of two repeated experiments are shown.

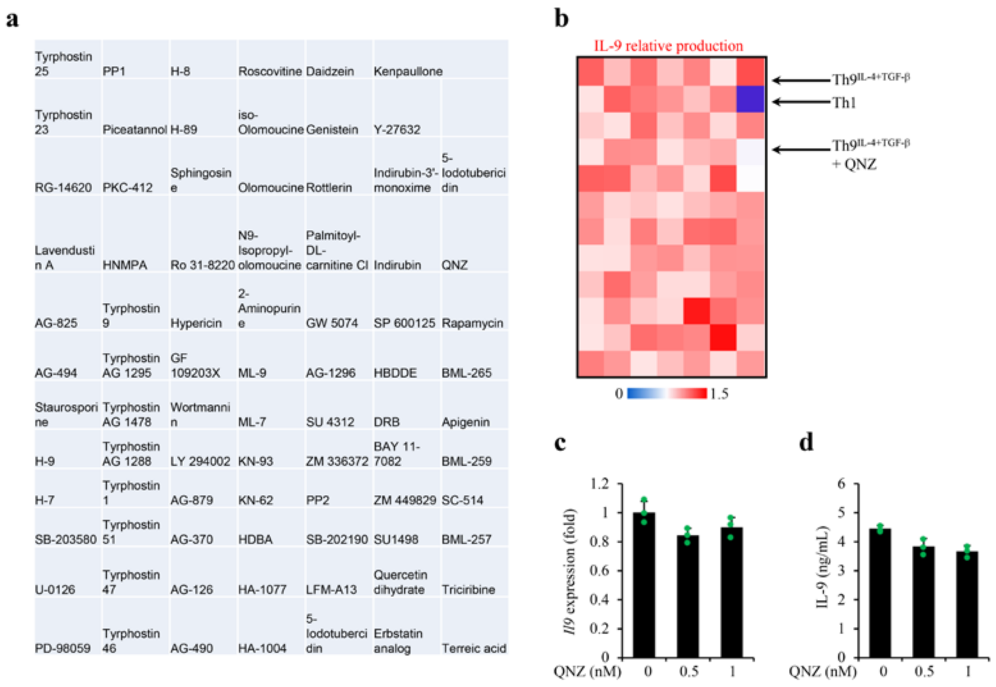

Tyrphostin 25	PP1	H-8	Roscovitine	Daidzein	Kenpaullone	
Tyrphostin 23	Piceatannol	H-89	iso-Olomoucine	Genistein	Y-27632	
RG-14620	PKC-412	Sphingosine	Olomoucine	Rottlerin	Indirubin-3'-monoxime	5-Iodotubercidin
Lavendustin A	HNMPA	Ro 31-8220	N9-Isopropyl-olomoucine	Palmitoyl-DL-carnitine Cl	Indirubin	QNZ
AG-825	Tyrphostin 9	Hypericin	2-Aminopurine	GW 5074	SP 600125	Rapamycin
AG-494	Tyrphostin AG 1295	GF 109203X	ML-9	AG-1296	HBDDE	BML-265
Staurosporine	Tyrphostin AG 1478	Wortmannin	ML-7	SU 4312	DRB	Apigenin
H-9	Tyrphostin AG 1288	LY 294002	KN-93	ZM 336372	BAY 11-7082	BML-259
H-7	Tyrphostin 1	AG-879	KN-62	PP2	ZM 449829	SC-514
SB-203580	Tyrphostin 51	AG-370	HDBA	SB-202190	SU1498	BML-257
U-0126	Tyrphostin 47	AG-126	HA-1077	LFM-A13	Quercetin dihydrate	Triciribine
PD-98059	Tyrphostin 46	AG-490	HA-1004	5-Iodotubercidin	Erbstatin analog	Terreic acid

Supplementary Figure 10. The function of NF-κB pathway on Th9$^{IL\text{-}4+TGF\text{-}\beta}$ cell differentiation. (a) The inhibitor labels of Kinase Inhibitor library. (b) IL-9 production was measured by ELISA in the supernatants of *in vitro* differentiated T cells. Shown is the heatmap of IL-9 relative production in the supernatants of Th1, Th9$^{IL\text{-}4+TGF\text{-}\beta}$ cells and inhibitor-treated Th9$^{IL\text{-}4+TGF\text{-}\beta}$ cells. (c and d) RT-PCR analysis of *Il9* transcriptional level (c) and ELISA of IL-9 production in the supernatants (d) of QNZ-treated Th9$^{IL\text{-}4+TGF\text{-}\beta}$ cells at indicated concentrations. QNZ, NF-κB pathway inhibitor. Data are mean ± SD (polarized *in vitro* for 3 days, n = 3). Representative results from one of two repeated experiments are shown.

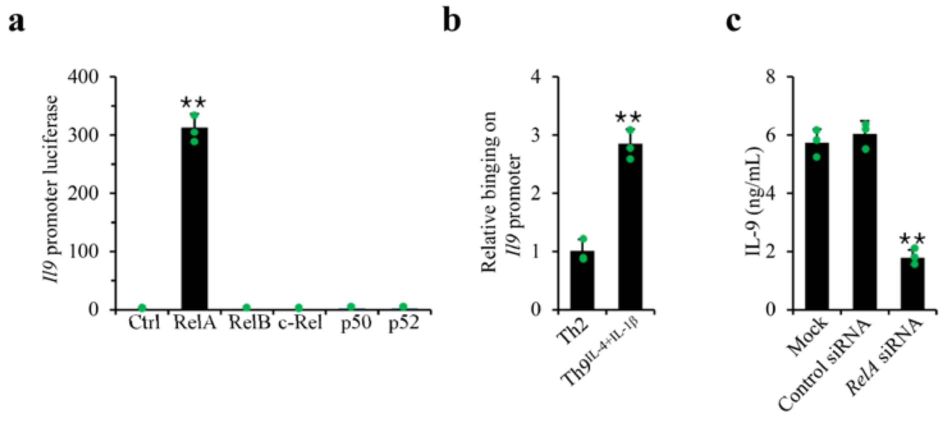

Supplementary Figure 11. The function of NF-κB family member RelA on IL-9 production in Th9$^{\text{IL-4+IL-1}\beta}$ cells. (a) Luciferase reporter assay for the activation of *Il9* promoter in 293T cells. (b) ChIP analysis of the binding of RelA at the *Il9* promoter in Th2 cells and Th9$^{\text{IL-4+IL-1}\beta}$ cells. (c) ELISA of IL-9 production in the supernatants of Th9$^{\text{IL-4+IL-1}\beta}$ cells treated by Mock or Control siRNA or RelA siRNA. Data are mean ± SD (polarized in vitro for 3 days, n = 3). **P <0.01, compared with others, Student's *t*-test. Representative results from one of two repeated experiments are shown.

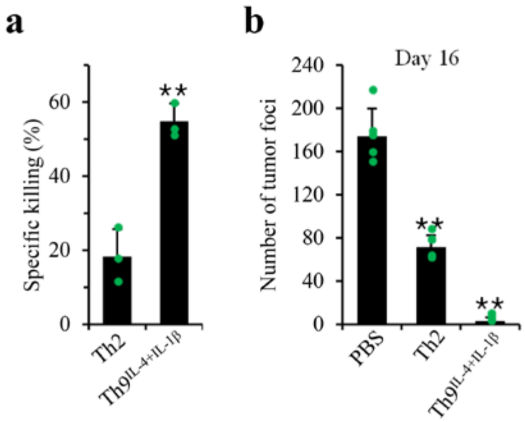

Supplementary Figure 12. Th9$^{IL-4+IL-1β}$ cells exhibited a greater anti-tumor capacity than Th2 cells. (a) Specific killing capacity of TRP-1 Th2 cells or Th9$^{IL-4+IL-1β}$ cells were performed against CFSE^{high}-B16 tumor cells as target cells (CFSE^{low}-MC38 tumor cells were used as a non-target control). An E:T ratio of 10:1 was used, and specific killing was determined after 48h of coculture (n = 3). (b) C57BL/6 mice were challenged with $1×10^5$ B16 cells delivered intravenously. $1 × 10^6$ TRP-1-specific Th2 cells or Th9$^{IL-4+IL-1β}$ cells were transferred on day 6 after tumor challenge, tumor foci in the lung were counted on day 16 after tumor inoculation (n = 5 mice/group). Data are mean ± SD; $**P < 0.01$, compared with other groups, Student's *t*-test. Representative results from one of two repeated experiments are shown.
