# Supplementary material for: IL-4 together with IL-1β induces antitumor Th9 cell differentiation in the absence of TGF-β signaling
Source: Nat Commun. 2019 Mar 26;10:1376. doi: 10.1038/s41467-019-09401-9 (PMC6435687; doi:10.1038/s41467-019-09401-9)
Supplement: Supplementary file 2 — Reporting Summary [file 41467_2019_9401_MOESM2_ESM.pdf]

nature research

Corresponding author(s): Yong Lu

Last updated by author(s): 2019.02.23

Reporting Summary

Nature Research wishes to improve the reproducibility of the work that we publish. This form provides structure for consistency and transparency in reporting. For further information on Nature Research policies, see Authors & Referees and the Editorial Policy Checklist.

Statistics

For all statistical analyses, confirm that the following items are present in the figure legend, table legend, main text, or Methods section.

n/a	Confirmed	
☐	☒	The exact sample size (*n*) for each experimental group/condition, given as a discrete number and unit of measurement
☐	☒	A statement on whether measurements were taken from distinct samples or whether the same sample was measured repeatedly
☐	☒	The statistical test(s) used AND whether they are one- or two-sided<br>*Only common tests should be described solely by name; describe more complex techniques in the Methods section.*
☒	☐	A description of all covariates tested
☒	☐	A description of any assumptions or corrections, such as tests of normality and adjustment for multiple comparisons
☐	☒	A full description of the statistical parameters including central tendency (e.g. means) or other basic estimates (e.g. regression coefficient) AND variation (e.g. standard deviation) or associated estimates of uncertainty (e.g. confidence intervals)
☒	☐	For null hypothesis testing, the test statistic (e.g. *F*, *t*, *r*) with confidence intervals, effect sizes, degrees of freedom and *P* value noted<br>*Give P values as exact values whenever suitable.*
☒	☐	For Bayesian analysis, information on the choice of priors and Markov chain Monte Carlo settings
☒	☐	For hierarchical and complex designs, identification of the appropriate level for tests and full reporting of outcomes
☐	☒	Estimates of effect sizes (e.g. Cohen's *d*, Pearson's *r*), indicating how they were calculated

Our web collection on statistics for biologists contains articles on many of the points above.

Software and code

Policy information about availability of computer code

Data collection	BD FACSDiva software
Data analysis	Flowjo 10

For manuscripts utilizing custom algorithms or software that are central to the research but not yet described in published literature, software must be made available to editors/reviewers. We strongly encourage code deposition in a community repository (e.g. GitHub). See the Nature Research guidelines for submitting code & software for further information.

Data

Policy information about availability of data

All manuscripts must include a data availability statement. This statement should provide the following information, where applicable:

- Accession codes, unique identifiers, or web links for publicly available datasets
- A list of figures that have associated raw data
- A description of any restrictions on data availability

The microarray data reported in this paper is deposited in the Gene Expression Omnibus (GEO) database under accession number GSE123624. The data that support the findings of this study are available from the corresponding author upon reasonable request.

Field-specific reporting

Please select the one below that is the best fit for your research. If you are not sure, read the appropriate sections before making your selection.

☒ Life sciences ☐ Behavioural & social sciences ☐ Ecological, evolutionary & environmental sciences

Life sciences study design

All studies must disclose on these points even when the disclosure is negative.

Sample size	No sample-size calculations were performed. Sample size was determined to be adequate based on the magnitude and consistency of measurable differences between groups.
Data exclusions	No data were excluded.
Replication	Experimental findings were highly reproducible each time
Randomization	No randomization of mice. Mice analyzed were litter mates and sex-matched whenever possible.
Blinding	The investigators were blinded to allocation during experiments and outcome assessment.

Reporting for specific materials, systems and methods

We require information from authors about some types of materials, experimental systems and methods used in many studies. Here, indicate whether each material, system or method listed is relevant to your study. If you are not sure if a list item applies to your research, read the appropriate section before selecting a response.

Materials & experimental systems

n/a	Involved in the study	
☐	☒	Antibodies
☐	☒	Eukaryotic cell lines
☒	☐	Palaeontology
☐	☒	Animals and other organisms
☒	☐	Human research participants
☒	☐	Clinical data

Methods

n/a	Involved in the study	
☒	☐	ChIP-seq
☐	☒	Flow cytometry
☒	☐	MRI-based neuroimaging

Antibodies

Antibodies used

IL-4-neutralizing antibody (clone 11B11) form BioXcell
IFN-γ-neutralizing antibody (clone XMG1.2) from BioXcell
IL-9-neutralizing antibody (clone 9C1) from BioXcell
CD4 depletion antibody (clone GK1.5) from BioXcell
TGF-β neutralizing antibody (clone 1D.11.16.8) from BioXcell
αp-Smad-3 flow antibody (clone O72-670) from BD
αp-Smad-5 flow antibody (clone 41D10) from CST
αCD4 flow antibody (clone RM4-4) from ThermoFisher
αIL-9 flow antibody (clone RM9A4) from ThermoFisher
αIL-4 flow antibody (clone 11b11) from ThermoFisher
αIL-10 flow antibody (clone JES5-16E3) from ThermoFisher
αIL-13 flow antibody (clone eBio13A) from ThermoFisher

Validation

All detailed validation information of these antibody can be obtained on the website.

Eukaryotic cell lines

Policy information about cell lines

Cell line source(s)	B16 melanoma cell line was purchased from ATCC, MC-38 cell line was a gift from Dr.Patrick Hwu, MD Anderson Cancer Center.
Authentication	None of the cell lines used were authenticated.
Mycoplasma contamination	All cell lines were tested negative for mycoplasma contamination.
Commonly misidentified lines (See ICLAC register)	No commonly misidentified cell lines were used.

Animals and other organisms

Policy information about studies involving animals; ARRIVE guidelines recommended for reporting animal research

Laboratory animals	C57BL/6, B6.Cg-Tg(Cd4-TGFBR2)16Flv/J, B6.129S2-Irf1tm1Mak/J, B6.Cg-Tg(TcraTcrb)425Cbn/J, B6.129S7-Il1r1tm1Imx/J and B6.Cg-Rag1tm1Mom Tyrp1B-w Tg(Tcra,Tcrb)9Rest/J mice were purchased from The Jackson Laboratory.
Wild animals	This study does not involve wild animals.
Field-collected samples	This study does not involve field-collected samples.
Ethics oversight	All experiments complied with protocols approved by the Institutional Animal Care and Use Committee at the Wake Forest School of Medicine.

Note that full information on the approval of the study protocol must also be provided in the manuscript.

Flow Cytometry

Plots

Confirm that:

- [ ] The axis labels state the marker and fluorochrome used (e.g. CD4-FITC).
- [x] The axis scales are clearly visible. Include numbers along axes only for bottom left plot of group (a 'group' is an analysis of identical markers).
- [x] All plots are contour plots with outliers or pseudocolor plots.
- [x] A numerical value for number of cells or percentage (with statistics) is provided.

Methodology

Sample preparation	Sample preparation listed in Methods.
Instrument	BD LSRFortessa X-20 Cell Analyzer.
Software	Flowjo 10.
Cell population abundance	More than 90%.
Gating strategy	Debris was first excluded by a morphology gate based on FSC-A and SSC-A. Then, we gated on LIVE/DEAD- CD4+ cells to exclude dead cells.

- [x] Tick this box to confirm that a figure exemplifying the gating strategy is provided in the Supplementary Information.
