# Supplementary material for: IL-4 together with IL-1β induces antitumor Th9 cell differentiation in the absence of TGF-β signaling
Source: Nat Commun. 2019 Mar 26;10:1376. doi: 10.1038/s41467-019-09401-9 (PMC6435687; doi:10.1038/s41467-019-09401-9)
Supplement: Supplementary file 3 — Description of Additional Supplementary Files [file 41467_2019_9401_MOESM3_ESM.pdf]

Description of Additional Supplementary Files

File Name: Supplementary Data 1

Description: Gene list of cluster 2 in Fig. 2

File Name: Supplementary Data 2

Description: Gene list of cluster 4 in Fig. 2

File Name: Supplementary Data 3

Description: Gene list of cluster 6 in Fig. 2
